# Desirable Components for a Customized, Home-Based, Digital Care-Management App for Children and Young People With Long-Term, Chronic Conditions: A Qualitative Exploration

**DOI:** 10.2196/jmir.7760

**Published:** 2017-07-04

**Authors:** Ruth Nightingale, Andrew Hall, Carole Gelder, Simone Friedl, Eileen Brennan, Veronica Swallow

**Affiliations:** ^1^ Great Ormond Street Hospital for Children NHS Foundation Trust London United Kingdom; ^2^ School of Healthcare University of Leeds Leeds United Kingdom; ^3^ School of Health Sciences University of Manchester Manchester United Kingdom; ^4^ Leeds Children's Hospital Leeds Teaching Hospitals NHS Trust Leeds United Kingdom; ^5^ Charles Sturt University Bathurst Australia

**Keywords:** child, adolescent, long-term condition, chronic condition, self-management, self-care, mobile apps, apps, qualitative

## Abstract

**Background:**

Mobile apps for mobile phones and tablet devices are widely used by children and young people aged 0-18 years with long-term health conditions, such as chronic kidney disease (CKD), and their healthy peers for social networking or gaming. They are also poised to become a major source of health guidance. However, app development processes that are coproduced, rigorously developed, and evaluated to provide tailored, condition-specific, practical advice on day-to-day care management are seldom systematic or sufficiently described to enable replication. Furthermore, attempts to extrapolate to the real world are hampered by a poor understanding of the effects of key elements of app components. Therefore, effective and cost-effective novel, digital apps that will effectively and safely support care management are critical and timely. To inform development of such an app for children with CKD, a user requirements-gathering exercise was first needed.

**Objective:**

To explore the views of children with CKD, their parents, and health care professionals to inform future development of a child-focused, care-management app.

**Methods:**

Using age- and developmentally appropriate methods, we interviewed 36 participants: 5-10-year-olds (n=6), 11-14-year-olds (n=6), 15-18-year-olds (n=5), mothers (n=10), fathers (n=2), and health care professionals (n=7). Data were analyzed using Framework Analysis and behavior change theories.

**Results:**

Of the 27 interviews, 19 (70%) interviews were individual and 8 (30%) were joint—5 out of 8 (63%) joint interviews were with a child or young person and their parent, 1 out of 8 (13%) were with a child and both parents, and 2 out of 8 (25%) were with 2 professionals. Three key themes emerged to inform development of a software requirement specification for a future home-based, digital care-management app intervention: (1) Gaps in current online information and support, (2) Difficulties experienced by children with a long-term condition, and (3) Suggestions for a digital care-management app. Reported gaps included the fact that current online information is not usually appropriate for children as it is “dry” and “boring,” could be “scary,” and was either hard to understand or not relevant to individuals’ circumstances. For children, searching online was much less accessible than using a professional-endorsed mobile app. Children also reported difficulty explaining their condition to others, maintaining treatment adherence, coping with feeling isolated, and with trying to live a “normal” life. There was recognition that a developmentally appropriate, CKD-specific app could support the process of explaining the condition to healthy peers, reducing isolation, adhering to care-management plans, and living a “normal” life. Participants recommended a range of media and content to include in a tailored, interactive, age- and developmentally appropriate app. For example, the user would be able to enter their age and diagnosis so that only age-appropriate and condition-specific content is displayed.

**Conclusions:**

Future development of a digital app that meets the identified information and support needs and preferences of children with CKD will maximize its utility, thereby augmenting CKD caregiving and optimizing outcomes.

## Introduction

The number of children and young people (children) with long-term/chronic health conditions is increasing and their information and support needs vary according to age and developmental stage. Providing appropriate information to children can promote better emotional health, less distress during treatments, greater satisfaction with health care, and optimal self-management and autonomy, which become crucial as they transfer to adult services [1].

Most children experience challenges throughout childhood and the transition into adulthood, but those with long-term conditions can experience additional challenges due to condition-specific needs. The Internet and mobile apps are increasingly used to communicate health-related information, but few websites or apps target children with long-term conditions; those that exist do so with variable quality and reliability [2]. A growing volume of literature on the self-management support needs of 5-18-year-olds with long-term conditions [1,3,4] indicates poor outcomes compared to other patient groups [5]. Those with chronic kidney disease (CKD) are particularly vulnerable due to complex medical and dietary regimens [6-8] and on transfer to adult services, treatment adherence often diminishes [9,10]. As CKD is progressive, poor adherence can lead to renal failure, which is fatal without renal replacement therapies [11,12]. Apps for mobile phones and tablet devices are now poised to become a major source of psychoeducational health guidance. However, to our knowledge, there are few coproduced, rigorously developed, evaluated apps providing tailored, CKD-specific, practical advice on day-to-day care management [13-20] and development processes for those that do exist are not systematic. Therefore, effective novel, digital solutions to guide child, young person, and parent consumers toward effective apps are critical and timely [14,21-29].

Acquiring the clinical skills and knowledge to manage treatment regimens effectively is a key factor in competent self- management. Therefore, robust, user-led, well-developed information and support interventions are needed that address children’s identified needs and preferences and that are supplemented by parents’ and health care professionals’ (professionals) views on what is realistic and achievable. The first author (RN), therefore, consulted with The Young People’s Advisory Group that she coleads at a UK children’s hospital. All group members reported using the Internet to find health information, were aware that websites varied in regards to accuracy and quality, and confirmed a need for research to develop and test reliable child-friendly apps for long-term conditions. Members thought that an app providing information about managing CKD, wider health education, vocational and social issues, and opportunities for safe interaction with others living with CKD could be very valuable.

A recent systematic review of the effectiveness of mobile apps for 10-24-year-olds with physical long-term conditions— published by authors of this manuscript—found a dearth of rigorously developed and evaluated user-led apps for this population [14]. Therefore, this study aimed to explore the views of children with CKD, their parents, and key professionals to inform the future development of a digital care-management app.

## Methods

The research aim of this study is to begin development and evaluation of an interactive child-led app to support home-based CKD management.

### Objectives

The first objective of this study is to determine the desirable components for a CKD- and child-focused app. The second objective of this study is to complete the theoretical modeling stage of our phased approach toward development and evaluation of a CKD-specific, home-based, digital care- management app.

### Methodology

Our study was founded on a key objective of the *life-stage approach* to UK policies for children with long-term conditions [30], which states that every child should have access to developmentally appropriate services. This includes resources that can be accessed easily, confidentially, at no cost to individuals, and in varied settings. We used the Medical Research Council (MRC) complex intervention development and evaluation framework to guide our study [31,32].

### Study Design and Sampling

A qualitative design was adopted that often uses small sample sizes to explore participants’ beliefs and practices within their natural context, rather than aiming to produce generalizable findings from large samples [33]. In two UK pediatric kidney units, participants were purposively sampled and included children with CKD and their parents. To achieve maximum variation regarding the children’s age, developmental stage, ethnicity, and sex, we aimed to recruit 6-8 participants from each of the following groups: 5-10-year-olds, 11-14-year-olds, 15-18-year-olds, and parents or carers of children with CKD.

Building on our prior work on *distributed expertise* [34], we aimed to invite 6-8 professionals (eg, clinical psychologists, dieticians, doctors, nurses, social workers, and play specialists) with experience in supporting families with CKD. Potential participants were identified by our two local principal investigators (SF and EB), who work clinically with children with CKD and their families. Once verbal consent was gained for the researcher (RN) to contact potential participants, age and developmentally appropriate written information was provided.

### Data Collection

Using a combination of semistructured individual or focus group interviews, depending on the individuals’ preferences [33,35], we collected data in child-friendly settings, such as children’s hospital-based venues or patients’ homes. Where children were interviewed jointly with their parents, we emphasized at the start of each interview that we would initially focus on the child’s views; therefore, all questions were directed at the child first, using developmentally appropriate language. We found that in some cases, the presence of the parent facilitated the child to share their views; for example, they would “translate” the researcher’s question to make it more relevant to their child, or prompt further response by using pertinent examples. Interviews were supported by topic guides that were developed by the research team in collaboration with members of our virtual research advisory group; these were based on our previous research and on behavior change theories.

We used age- and developmentally appropriate, technology- supported methods to explore children’s views on the strengths and limitations of existing Web and mobile resources relating to CKD or general health issues. For example, we demonstrated existing Web resources, such as websites and mobile apps, focused on children with CKD and other long-term conditions to facilitate discussion about desirable components, potential barriers and facilitators to using Web and/or mobile apps, preferred designs, functionality, and levels of interaction. Creative methods were used, in particular with 5-10-year-olds who may have more difficulty expressing themselves verbally [36,37]. For example, we used drawing to find out what impact CKD has on a “typical” day and to encourage child participants to express their ideas for what they thought should be included in an app. This activity was offered to all children who were either chronologically or developmentally in the 5-10-year-old group and 2 participants chose to draw; 1 drew himself in the hospital and 1 produced images of games to include in the app. Advantages of these types of research techniques include the following: engaging children and encouraging their participation; relevance to children’s own styles of expression and interest in images; and creative tasks that can help sustain attention and interest, going beyond standard ways of answering questions and leaving time for participants to reflect and think [38]. The 2 participants who created images were asked to explain these to the researcher; these conversations were recorded, transcribed, and analyzed in conjunction with the images produced.

Interviews with parents and professionals were also technology supported and focused on their views on existing apps, gaps in existing resources for children, and suggestions for desirable components for a child-focused, CKD-specific, information and support app. We explored participants’ views on content, patient information services, peer-to-peer interactions, security, and data sharing.

Interventions based on behavior change theories are reported to be more effective than those lacking a theoretical basis. One of the most influential behavior change theories in health apps and Internet resources is Bandura’s social cognitive theory, an interpersonal theory that covers both determinants of behavior and methods for behavior change [17,39,40]. A major determinant of behavior that social cognitive theory describes is self-efficacy (eg, feeling self-confidence in managing the condition). This approach proposes that an individual’s belief in their own self-efficacy comes from the following: mastery experiences (eg, improved communication skills about their condition with family, friends, and professionals, or learning by practicing clinical skills); vicarious experiences (eg, observing how professionals effectively deliver clinical skills or how peers with a similar condition enact healthy behaviors); verbal persuasion (eg, reading about or listening to others explain how they successfully manage certain self-management experiences); and physiological or affective experiences (eg, decreased worry about themselves when they have mastered ways to deal with negative emotions relating to their condition). Efficacy in self-management enhances adoption and maintenance of positive health habits such as adherence to treatment regimens. In the context of developing an information and support app, the theory provided a schema for how self-efficacy beliefs about self-management could potentially be promoted by the app. Therefore, because of the need for individual support and because rigorously developed apps are more likely than not to improve users’ self-efficacy, in our analysis we drew upon social cognitive theory and self-efficacy [41].

### Data Analysis

Data were analyzed using Framework [33,42], a recognized, systematic method for handling large amounts of qualitative data. Framework builds findings by moving back and forth between collecting data, identifying themes, coding and labeling data, identifying categories, detecting patterns, and seeking possible interpretations. Independent reviews of data samples were discussed by the authors (RN, AH, CG, and VS) until a consensus was achieved. We interviewed participants until reaching theoretical saturation [33].

### Ethical Considerations

For children, age- and developmentally appropriate written and verbal information was provided. Participants under 16 years of age provided assent and their parents provided consent for their child’s participation. Approval was obtained from the Health Research Authority, a National Health Service (NHS) Research Ethics Committee (reference No. 16/NW/0227), and the NHS Trust Research and Development Departments.

### Patient and Public Involvement

Patient and public involvement (PPI) was instrumental in our study. INVOLVE defines PPI as “research being carried out ‘with’ or ‘by’ members of the public rather than ‘to,’ ‘about,’ or ‘for’ them,” as seen on page 6 of its Briefing Notes for Researchers [43]. We used this definition to establish a virtual research advisory group comprising young people with CKD and parents of children with CKD. Group members advised on the following: study management (parents were active members of the study steering group), interview topic guides, Web resources used during interviews, data analysis and dissemination.

## Results

### Overview

A total of 36 participants were interviewed. Table 1 provides information about the participants.

**Figure 1:**
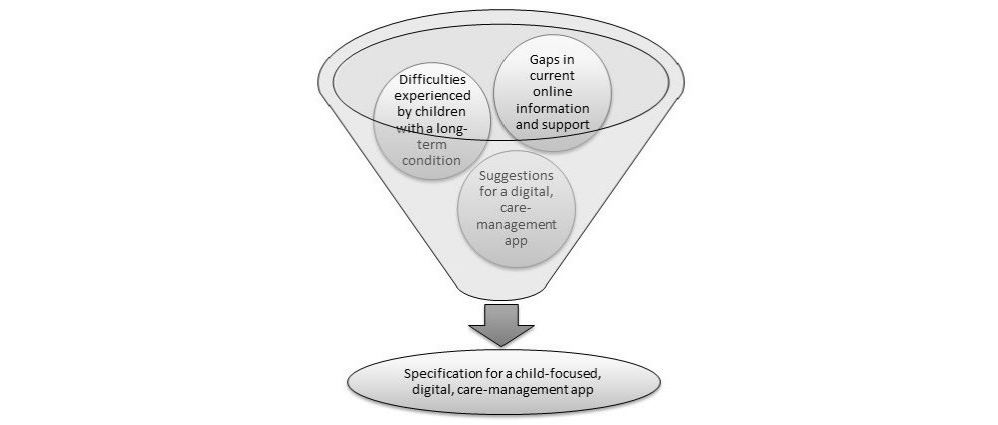
Overview of study findings.

Of the 27 interviews conducted, 19 (70%) were individual and 8 (30%) were joint—5 out of 8 (63%) joint interviews were with a child or young person and their parent, 1 out of 8 (13%) were with a child and both parents, and 2 out of 8 (25%) were with 2 professionals. For convenience, 25 out of 27 (93%) interviews were face-to-face and 2 out of 27 (7%) were telephone interviews. Interviews lasted between 8 and 55 minutes, were digitally recorded, and later transcribed verbatim. The first author (RN) conducted 22 of the 27 (81%) interviews—3 jointly with another author (CG)—and the last author (VS) conducted 5 out of 27 (19%) interviews. Analysis identified three key themes to inform development of a software requirement specification [44]: (1) Gaps in current online information and support, (2) Difficulties experienced by children with a long-term condition, and (3) Suggestions for a digital care-management app (see Figure 1).

**Table 1:** Participant characteristics.

Participant characteristics		Children aged 5-10 years (n=6), n (%)	Young people aged 11-14 years (n=6), n (%)	Young people aged 15-18 years (n=5), n (%)	Parents (n=12), n (%)	Professionals (n=7), n (%)
**Gender**						
	Female	3 (50)	3 (50)	2 (40)	10 (83)	N/A^a^
	Male	3 (50)	3 (50)	3 (60)	2 (17)	N/A
**Ethnicity**						
	White British	4 (67)	5 (83)	4 (80)	N/A	N/A
	Other	2 (33)	1 (17)	1 (20)	N/A	N/A
**Profession**						
	Doctor	N/A	N/A	N/A	N/A	1 (14)
	Nurse	N/A	N/A	N/A	N/A	2 (29)
	Play specialist	N/A	N/A	N/A	N/A	1 (14)
	Social worker	N/A	N/A	N/A	N/A	2 (29)
	Therapist	N/A	N/A	N/A	N/A	1 (14)

^a^N/A: not applicable.

### Gaps in Current Information and Support

Child and parent participants advised that they obtained the majority of their information and support from professionals in the renal multidisciplinary team responsible for their care. Those who looked online for information visited a range of websites including the following: NHS Choices, infoKID, and the National Kidney Foundation. Reported gaps in current online information and support were related to issues such as content, trustworthiness, child friendliness, and accessibility.

Young people, professionals, and parents with older children thought the information on websites was quite basic, lacked detail, and was unhelpful in developing self-confidence in managing the condition. One parent stated the following:

...a couple of times he [son] has told me he's looked things up on the Internet. But I try to dissuade him from doing that because I don't think you ever get any real reassurance, because it's so wide, isn't it, the Internet, it's not tailored to specifics.

Parent A of 13-year-old child

All participant groups concluded that to gain information that was specific to the child’s situation, the most helpful source of information was the professionals involved in their care. They recognized that professionals could provide tailored information that was relevant, timely, and individual specific, but online information could be “scary,” as it was either hard to understand or not relevant to the individual’s circumstances.

Concern was expressed about whether websites contained accurate information and, therefore, whether they could be relied upon to promote mastery, although those from recognized organizations (eg, NHS Choices and the National Kidney Foundation) were viewed as more trustworthy, as this quotation illustrates:

Most of the sites regarding stuff like diet are like forums, so anyone can post, so there’s not really that much reliability...the Kidney Foundation or something, that’s pretty reliable obviously ’cause it’s a government website, so I use that mostly.

Young person, aged 17 years

Current online information was described as not appropriate for children, as it is “dry,” “boring,” or as one professional said:

...the written information’s a bit dull, I think a lot of it’s been written by doctors and it’s not hugely fit for purpose, it’s not interactive, you know. You look at kids now and the way they learn is through iPads and apps, and it’s all of this isn’t it? And I think we’re [professionals] quite behind on that, but it’s just trying to find the time to develop that.

Professional

Parent and young people participants expressed concerns about the accessibility of online information, reporting difficulties with searching for, and finding, information. Though some reported that professionals had recommended specific websites, for others the preference was to ask their professional, rather than “trawl” through information online. For some young people, searching online was considered much less accessible than using a mobile app:

I don’t really go online to look about kidney things, so I think if there was an app because people are very “oh my phone, oh look, have you seen this app?” these days, so it would be quite accessible.

Young person A, aged 11 years

### Difficulties Experienced by Children With a Long-Term Condition

All participant groups described difficulties experienced by children living with CKD. This included the following: the need for information to support self-care and, therefore, promote feelings of self-efficacy; explaining their condition to others; treatment adherence; feeling isolated; and living a “normal” life.

All participants viewed the provision of information and support to children and their families as essential. Nevertheless, it could be difficult to understand some information provided by professionals, especially at stressful times such as diagnosis, or when treatments changed. Some parents found it challenging to ensure their child had information they could understand, especially if their child was diagnosed when young or became unwell suddenly.

The difficulties associated with explaining their condition to others, including friends and extended family members, were discussed by many child, young person, and parent participants. This appeared particularly resonant for families where the condition is invisible, in that there are no outward effects on the child, such as CKD. Child and young person participants talked of describing their condition, to whom they disclosed this information, and others’ responses:

I only have one friend, but she always asks how I am and everything, and I tell her, because she understands what I’ve been through. But the only thing is, I only tell her, but I think she keeps telling everyone, when I say at the end of our conversation, “please don’t tell anyone else, because they tell everyone else as well.”

Young person B, aged 11 years

There was recognition that a developmentally appropriate, CKD-specific app could support the process of explaining the condition to others.

All participant groups talked about the complex and often unpleasant treatments needed by children with CKD, including the need to master medication, dietary and fluid restrictions or targets, catheterization, and dialysis. There was discussion around the difficulties of following treatment regimens, which seemed to result from forgetting, deciding not to adhere, or misunderstanding due to changes in treatment, as illustrated by this interview excerpt from a young person who had recently received a kidney transplant:

Because a lot of children think, like, before [being diagnosed with CKD] I was allowed to eat that; now you tell me I’m not allowed to eat it. And then after they’ve had an operation [kidney transplant] and you tell them, you can eat it now, they don’t understand why they weren’t allowed to eat it, and now they can eat it.

Young person C, aged 11 years

Feeling isolated due to having CKD was discussed by some children and parents. Some children talked of the difficulty of not knowing anyone else with CKD and feeling like they were “the only one.” Many said they would value being able to connect with others with a similar condition.

I don't really like looking at the websites...because it reminds me of how much I'm different from all the rest of my friends.

Child, aged 8 years

Though some children felt different from their peers, the importance of living a “normal” life, despite having CKD, was explored during interviews. The impact of having CKD was discussed, including missing school to attend hospital appointments and manage treatment regimens. Discussing the techniques used to cope, such as keeping active and avoiding thinking about how the condition may affect them in the future, meant many children felt they were able to lead a “normal” life. Parent participants also viewed this as important, as this quotation illustrates:

...he'll have his transplant and then he'll move on and have a relatively normal life. And that's what I want for him as a parent. So I want him to feel like he understands his condition and everything, but I don't want it to define him. I want it to just be something that's part of him.

Parent A of 13-year-old child

### Suggestions for a Digital Care-Management App

Participants from all groups recommended an interactive, age- and developmentally appropriate care-management app, whereby the user could enter their age and their diagnosis, so only age- and condition-specific content is displayed.

There was some discussion about whether a mobile app or a website would be preferable. Participants who preferred a website thought the content would be more suitable for a webpage layout and were concerned about the potential size of the app on their device. However, many participants thought an app would be preferable as they were more accessible and interactive:

I think an app would probably be better, rather than going on a website to do it, because apps are more convenient. You don’t have to type anything up and you can just click on it.

Young person, aged 16 years

Other key features suggested were endorsement of the app by renal professionals, as it was important for children and their families to know the information in the app was accurate and trustworthy. It was also thought important that the app be promoted as a supplement, not a replacement for individual advice and support from professionals. Participants from all groups suggested the app should emphasize the importance of consulting with professionals for information, advice, and support and the need to contact them with unanswered questions or concerns.

All participant groups suggested the app should contain information about the human body, with a particular focus on the kidneys and how they interact with other body systems. Information about kidney conditions, symptoms, and stages of kidney disease was thought useful, though some parent and professional participants expressed concerns that children viewing information about how their condition could progress could potentially view it as “scary.” It was also suggested that information about common tests and procedures should be included in the app to help children feel better prepared for such procedures. Additionally, participants from all groups requested information about different treatments for kidney disease, including medication, diet, dialysis, and transplant, as illustrated in the following quote:

The knowledge about the transplant process, and pictures, and she can look at it for herself, instead of me telling her, or somebody else telling her.

Parent B of 13-year-old child

It was thought this could help a child understand about their current treatment and could potentially help with learning about what the future may involve. Finally, information about the potential impact of having kidney disease on (1) physical well-being and (2) emotional well-being/mental health was viewed as important, while ensuring there is an emphasis on what children with CKD can still do and how to lead a normal life.

Participants from all groups suggested it would be beneficial if the app could be used to record information, including details of their treatment regimen (eg, medication, fluid target, and diet), appointments, and linkup with existing electronic patient records. This could promote treatment adherence by providing reminders and alarms, for example, for their medication and encourage children to record when treatment had been completed. This is illustrated in the following quote:

Some sort of planner on it, when their appointments are, little alerts for medication, that might help, especially as they’re beginning to transition, giving them a bit more independence.

Professional

Some professional participants suggested the app could also be used to record emotions and physical symptoms. It was suggested that professionals managing the children’s CKD could also review the recorded information; however, some professionals expressed concerns around this in case children thought professionals were monitoring this information and would respond if anything required immediate attention.

Information related to the vicarious experiences of other children regarding the consequences of adhering or not adhering to treatment (eg, medication, fluids, and diet) and other lifestyle choices (eg, smoking and drinking alcohol) was suggested by participants from young person, parent, and professional groups. It was thought this could help children make “smarter” decisions as they would have a clearer understanding of the consequences of choosing not to follow advice from professionals. The idea of decision-making tools was discussed, as illustrated by this quotation from a young person who discussed the benefits of being able to use her phone to scan barcodes on food products to identify the ingredients:

I’m on a low-phosphate diet, and I have to be careful of things that have phosphate in them, sometimes you have to look at the packaging. But some companies don’t have to put in if it has phosphate, so it’s hard to know what you’re eating...If I was in a shop and I could, like, scan something, and if it told me what it had in it...and it could rule out if it has phosphate, and it could tell you what’s in it, and if it’s a bad thing, that you’re not allowed, it could be in red, so you know that it’s bad.

Young person, aged 13 years

Many participants, in particular children and parents of children under 13 years, thought games should be included in the app; for example, games with information about how the kidney or treatments work and incorporating elements of game design, such as scoring points and progressing through levels. It was suggested that gamification would encourage children to engage with the app and learn about their condition.

However, some participants expressed concerns around gamification; older teenagers thought games could work to engage younger children, but thought it unlikely that older teenagers would play a CKD-focused game. There was recognition of the addictive nature of many games and while some parent and professional participants thought this beneficial as a way to engage children, others expressed concerns as illustrated by this quotation:

I don’t think we want to be over-hip, (a) because I don’t think it would work and (b) because I think I don’t want to go so far down the gaming culture that...in a way that I think would be unhelpful for some young people.

Professional

There were suggestions that a safe, moderated forum where children with CKD could communicate with their peers would be beneficial, as it could provide access to vicarious experiences from other children similar to themselves who are succeeding in self-management; this could potentially increase their beliefs that they, too, can master self-management. The value of being able to interact, ask questions, and share experiences was recognized by many child and young person participants who sometimes felt isolated as a result of having CKD:

So this person can see that person and make them feel better about it, because people knowing that other people have it [CKD] makes them feel better.

Child, aged 10 years

Although children and professionals currently interact through a range of methods, including appointments, telephone, and email, some young person participants thought it would also help to be able to interact via the app. Suggestions included having live question-and-answer sessions and having professionals be able to access the children’s forum so they could contribute to discussions.

Hearing other children’s experiences of living with CKD was suggested by many participants from all groups as important to include in the app, for example, via blogs, vlogs, and photos to share the challenges of having CKD and adhering to treatment:

It’s not all about, “yes, it’s brilliant, I’m really relaxed here,” they’ve got to say something about the other sides to it, and then they can relate to it.

Parent B of 13-year-old child

Linking patient stories into other parts of the app that focused on supporting children to make “smarter” decisions was recommended as a way that could potentially encourage children to develop self-management skills. Some also thought that hearing about celebrities and/or young adults’ achievements despite having CKD could be useful.

Parent and professional participants thought the app could contain signposting and links to other relevant and trustworthy websites, such as infoKID, charities, and websites with information about available support, community and social activities, and research.

Finally, participants from all groups made suggestions about the design and usability of a digital care-management app, which are listed in Multimedia Appendix 1.

## Discussion

### Principal Findings

Few studies have sought the views of children with long-term conditions on the desired components of a child-friendly, information and support software app as part of a phased approach to development and evaluation of a complex intervention [31,32]. To our knowledge, this is the first study that has explored this issue with children living with CKD. However, although this study focused on children with CKD, the methodology used and many of our findings are potentially transferrable to other long-term conditions.

Our main finding is that due to important gaps in current information and support and the difficulties experienced by children with CKD, there is strong support among children, parents, and professionals for a CKD-specific, child-focused software app. This would complement information and support provided by professionals to enhance self-management and optimize high-quality care. Participants’ suggestions reflect their/their child’s age or developmental stage, so it is critical that the app be appropriate for a wide range of children and that there is flexibility in how it could be used.

Our findings correspond with reports in the literature about the importance of interventions that aim to improve general self-efficacy among children with long-term conditions, to enhance confidence and the ability to deal effectively with difficult and/or unexpected self-management events [45,46]. Self-efficacy is an individual’s judgment of his or her capability to accomplish a certain level of performance in a given set of skills. In preparation for future research to empirically optimize the app before taking the optimal version forward to a national confirmatory trial, we will also consider other determinants of behavior that are described by social cognitive theory, such as outcome expectations or a person’s judgment of the likely consequences of a behavior (eg, “When I follow the correct procedure for cleaning my central venous line catheter insertion site, I will prevent the introduction of infection.”). Bandura was explicit about the interrelation between outcome expectations and self-efficacy, claiming that judgments of ability to perform a behavior greatly influence expected outcomes. When a person is in a situation in which outcome expectations are positive and strong, but self-efficacy for that behavior is low, a situation of avoidance may occur and the individual is unlikely to attempt the behavior. Therefore, interventions that aim to improve general self-efficacy are expected to be beneficial.

Our findings also highlight a significant need for mobile phone games (ie, gamification) that aim to optimize self-efficacy and positively alter health behaviors in chronic disease self-management. This corresponds with a recent systematic review that reports the following: (1) few health apps currently employ gamification, (2) there is a wide variation in the use of behavior change theories and techniques, (3) this may limit potential to improve health outcomes, and (4) further research is required in this field [46].

### Software App Specification Development

As illustrated in Figure 1, our data were analyzed and used to develop a software requirement specification for a child-focused, home-based, digital care-management app. The emergent themes were mapped onto functional requirements within a functional specification document; this describes the externally visible behavior of a proposed software app and forms part of a systems requirement document—the basis for much software development. The interviews with participants were in effect treated as a detailed and rigorous user requirement-gathering exercise, and our analysis as a robust method to determine key desired components within a potential app. The interview data that was gathered and coded into themes provided the design and functionality detail for each of the components identified. As Goldsmith [47] notes, a software app is unlikely to be better than its requirements; often, the typical methods to gather information about what potential app users need and prefer tend to only deliver partial requirements, resulting in users’ needs frequently getting “lost in translation” when the app is designed.

In the emerging field of evidence-based app development for children with long-term conditions [48-50], a partial, misinterpreted or distorted understanding of children’s, parents’, and professionals’ requirements can lead to the development of health care apps that are suboptimal and not fit for purpose. While there is significant debate around the rigor and transferability of qualitative health research findings, in particular Framework Analysis as used in this study [33,51], little attention is paid to the following: (1) the growing need to use qualitative research methods to gather information about users’ requirements and (2) the development of integrated health apps for personal mobile devices for children in a participatory way [14]. Adopting a rigorous qualitative approach within a participatory and ethical research environment can only strengthen the effectiveness, functionality, quality, and efficient development of such apps.

Our study drew the discipline of qualitative research into the early stages of software development and produced a functional specification; next, it needs to draw methods from software development into the qualitative research space. From the functional specification, tools can be produced and used as objects to prompt further consideration and refinement of user needs; these tools can include use cases, which illustrate the interaction an app user will have with the software system to achieve a given task, and wireframes, a graphical representation of content and functionality of the app [52]. It is after this point that the additional elements of the full requirements specification can be considered, which include system requirements, technical requirements, constraints, assumptions, and acceptance criteria. Once an app is developed, it will undergo a rigorous and iterative process of testing and refinement as outlined by the MRC guidance on development and evaluation of complex interventions [31].

### Practice Implications

Professionals are responsible for empowering children with long-term conditions, such as CKD, and their parents to develop self-management skills. Good-quality information and support is essential to help children and parents understand and cope with the condition. However, professionals need to be mindful of individual families’ information needs and tailor further information provision and signposting to resources that are age and stage appropriate [6,35,53,54]. As illustrated in our study, families often need help to identify reliable sources of information online and to recognize the limitations of this information.

It would be beneficial if multidisciplinary teams are aware of the lived experience of children with long-term conditions and which additional sources of support are important; for example, social isolation has been highlighted as a problem by both children with CKD and their parents [55] who often live a long way from face-to-face professional support, thus necessitating supplementary digital support.

There is a clear need to address children’s information and support needs in ways that take account of individual and family circumstances and, therefore, enhances children’s adjustment to, and mastery of, the demands of living with a long-term condition. It is acknowledged that the methods used by professionals within the NHS to deliver high-quality information to children has not kept pace with advances in available technology [14].  Given the ubiquity of mobile phone use by children, developing online and mobile apps that support self-management and are used to complement professionals’ individualized advice is an important step in providing a range of interventions to support children with long-term conditions [56]. For example, in pediatric diabetes care, a number of online resources have been developed [57]; however, it is unclear how these apps were developed and whether their impact on self-management has been evaluated. There is also emerging evidence that the use of software apps could potentially engage patient groups, which professionals have struggled to reach with more traditional interventions, though further research is needed in this area [58].

### Strengths and Limitations

A key strength of our study was the participatory approach using age-appropriate and technology-supported interviews that included drawing and the use of existing software apps; this enabled us to interview children between the ages of 5 and 17 years. Ensuring interviews were a visual and interactive experience encouraged verbal interaction, particularly with 5-10-year-olds who may have more difficulties expressing themselves verbally and are more concrete in their thinking [59].

Additionally, PPI throughout the study brought a different perspective to our work and helped ensure its relevance to children with CKD and their families.

A final strength of our study was the multidisciplinary research team, which consisted of researchers, an educational technologist, and clinicians, including a clinical psychologist and nurses, with experience of working with children living with CKD and diabetes. Having this range of expertise and different viewpoints benefitted the study and enabled us to explore the transferability of our approach and findings to children living with other long-term conditions.

Our sample included children between the ages of 5 and 17 years and it was evident that children’s needs and preferences differ based on many factors, including their age, developmental stage, and individual preferences. However, due to our small sample size, we were only able to reach speculative conclusions regarding their needs and preferences. Further research to explore this issue would be beneficial as part of a phased approach to developing a child-focused software app to support self-management.

### Conclusions

Developing an evidence-based mobile app that meets the information and support needs of children with long-term conditions such as CKD will maximize its utility, thereby augmenting their ability to learn to confidently manage their condition and optimize their outcomes. Through working collaboratively with patients, parents, and professionals, and by employing a conceptual framework that explicitly acknowledges the importance of promoting children’s self-efficacy, we can offer new insights into the digital support and information needs of patients at different developmental stages. These insights highlight the gaps in current information and support, including that provided via mobile apps, the difficulties experienced by children with a long-term condition, and suggestions for a digital care-management app. This study has provided a responsive framework with which to further develop and evaluate digital app resources for children with CKD.

## Appendix

Multimedia Appendix 1 Suggestions for the design and usability of a digital care-management app.

## Abbreviations

CKD: chronic kidney disease
MRC: Medical Research Council
N/A: not applicable
NHS: National Health Service
PPI: patient and public involvement

## References

[1] (2014). Health for the World's Adolescents: A Second Chance in the Second Decade.

[2] McPherson AC, Gofine ML, Stinson J (2014). Seeing is believing? A mixed-methods study exploring the quality and perceived trustworthiness of online information about chronic conditions aimed at children and young people. Health Commun.

[3] Swallow V, Lambert H, Clarke C, Campbell S, Jacoby A (2008). Childhood chronic kidney disease: A longitudinal qualitative study of families learning to share management early in the trajectory. Patient Educ Couns.

[4] Anthony SJ, Hebert D, Todd L, Korus M, Langlois V, Pool R, Robinson LA, Williams A, Pollock-BarZiv SM (2010). Child and parental perspectives of multidimensional quality of life outcomes after kidney transplantation. Pediatr Transplant.

[5] Beard C (2013). From the Start: Engaging Young Adults With Long-Term Conditions in Their Care.

[6] Tjaden L, Tong A, Henning P, Groothoff J, Craig JC (2012). Children's experiences of dialysis: A systematic review of qualitative studies. Arch Dis Child.

[7] Warady BA, Chadha V (2007). Chronic kidney disease in children: The global perspective. Pediatr Nephrol.

[8] Marks SD (2007). How have the past 5 years of research changed clinical practice in paediatric nephrology?. Arch Dis Child.

[9] Colver A, Longwell S (2013). New understanding of adolescent brain development: Relevance to transitional healthcare for young people with long-term conditions. Arch Dis Child.

[10] Schwartz LA, Tuchman LK, Hobbie WL, Ginsberg JP (2011). A social-ecological model of readiness for transition to adult-oriented care for adolescents and young adults with chronic health conditions. Child Care Health Dev.

[11] (2005). The National Service Framework for Renal Services. Part Two: Chronic Kidney Disease, Acute Renal Failure and End of Life Care.

[12] (2009). Helping Adolescents and Young Adults With End Stage Renal Failure.

[13] Improved Clinical Effectiveness through Behavioural Research Group (ICEBeRG) (2006). Designing theoretically informed implementation interventions. Implement Sci.

[14] Majeed-Ariss R, Baildam E, Campbell M, Chieng A, Fallon D, Hall A, McDonagh JE, Stones SR, Thomson W, Swallow V (2015). Apps and adolescents: A systematic review of adolescents' use of mobile phone and tablet apps that support personal management of their chronic or long-term physical conditions. J Med Internet Res.

[15] Stinson JN, Lalloo C, Harris L, Isaac L, Campbell F, Brown S, Ruskin D, Gordon A, Galonski M, Pink LR, Buckley N, Henry JL, White M, Karim A (2014). iCanCope with Pain: User-centred design of a web- and mobile-based self-management program for youth with chronic pain based on identified health care needs. Pain Res Manag.

[16] Free C, Phillips G, Galli L, Watson L, Felix L, Edwards P, Patel V, Haines A (2013). The effectiveness of mobile-health technology-based health behaviour change or disease management interventions for health care consumers: A systematic review. PLoS Med.

[17] Vollmer DD, Fair K, Hong YA, Beaudoin CE, Pulczinski J, Ory MG (2015). Apps seeking theories: Results of a study on the use of health behavior change theories in cancer survivorship mobile apps. JMIR Mhealth Uhealth.

[18] Grundy QH, Wang Z, Bero LA (2016). Challenges in assessing mobile health app quality: A systematic review of prevalent and innovative methods. Am J Prev Med.

[19] Kratzke C, Cox C (2012). Smartphone technology and apps: Rapidly changing health promotion. Int Electron J Health Educ.

[20] Brannon EE, Cushing CC (2015). A systematic review: Is there an app for that? Translational science of pediatric behavior change for physical activity and dietary interventions. J Pediatr Psychol.

[21] Nightingale R, Friedl S, Swallow V (2015). Parents' learning needs and preferences when sharing management of their child's long-term/chronic condition: A systematic review. Patient Educ Couns.

[22] Swallow V, Macfadyen A, Santacroce SJ, Lambert H (2012). Fathers' contributions to the management of their child's long-term medical condition: A narrative review of the literature. Health Expect.

[23] Eiser C, Jenney M (2007). Measuring quality of life. Arch Dis Child.

[24] Gerson AC, Wentz A, Abraham AG, Mendley SR, Hooper SR, Butler RW, Gipson DS, Lande MB, Shinnar S, Moxey-Mims MM, Warady BA, Furth SL (2010). Health-related quality of life of children with mild to moderate chronic kidney disease. Pediatrics.

[25] Elwyn G, Frosch D, Thomson R, Joseph-Williams N, Lloyd A, Kinnersley P, Cording E, Tomson D, Dodd C, Rollnick S, Edwards A, Barry M (2012). Shared decision making: A model for clinical practice. J Gen Intern Med.

[26] Leeman J, Crandell JL, Lee A, Bai J, Sandelowski M, Knafl K (2016). Family functioning and the well-being of children with chronic conditions: A meta-analysis. Res Nurs Health.

[27] Knafl KA, Havill NL, Leeman J, Fleming L, Crandell JL, Sandelowski M (2017). The nature of family engagement in interventions for children with chronic conditions. West J Nurs Res.

[28] Sawyer SM, Afifi RA, Bearinger LH, Blakemore S, Dick B, Ezeh AC, Patton GC (2012). Adolescence: A foundation for future health. Lancet.

[29] Alderwick H, Robertson R, Appleby J, Dunn P, Maguire D (2015). Better Value in the NHS: The Role of Changes in Clinical Practice.

[30] (2013). Chief Medical Officer's Annual Report 2012. Our Children Deserve Better: Prevention Pays.

[31] (2008). Developing and Evaluating Complex Interventions: New Guidance.

[32] Richards DA, Hallberg IR (2015). Complex Interventions in Health: An Overview of Research Methods.

[33] Ritchie J, Lewis J, McNaughton Nicholls C, Ormston R (2014). Qualitative Research Practice: A Guide for Social Science Students and Researchers. 2nd edition.

[34] Swallow V, Smith T, Webb NJ, Wirz L, Qizalbash L, Brennan E, Birch A, Sinha MD, Krischock L, van der Voort J, King D, Lambert H, Milford DV, Crowther L, Saleem M, Lunn A, Williams J (2015). Distributed expertise: Qualitative study of a British network of multidisciplinary teams supporting parents of children with chronic kidney disease. Child Care Health Dev.

[35] Swallow V, Coad J, Macfadyen A, Nolan M, Hanson E, Grant G, Keady J (2007). User Participation Research in Health and Social Care: Voices, Values, and Evaluation.

[36] Bagnoli A, Clark A (2010). Focus groups with young people: A participatory approach to research planning. J Youth Stud.

[37] Bagnoli A (2009). Beyond the standard interview: The use of graphic elicitation and arts-based methods. Qual Res.

[38] Gauntlett D (2007). Creative Explorations: New Approaches to Identities and Audiences.

[39] Holloway A, Watson HE (2002). Role of self-efficacy and behaviour change. Int J Nurs Pract.

[40] Bandura A (1997). Self-Efficacy: The Exercise of Control.

[41] Bandura A (1998). Health promotion from the perspective of social cognitive theory. Psychol Health.

[42] Ward DJ, Furber C, Tierney S, Swallow V (2013). Using Framework Analysis in nursing research: A worked example. J Adv Nurs.

[43] Hayes H, Buckland S, Tarpey M (2012). Briefing Notes for Researchers: Involving the Public in NHS, Public Health, and Social Care Research.

[44] Kumar N, Zadgaonkar A, Shukla A (2013). Evolving a new software development life cycle model SDLC-2013 with client satisfaction. Int J Soft Comput Eng.

[45] Cramm JM, Strating MM, Roebroeck ME, Nieboer AP (2013). The importance of general self-efficacy for the quality of life of adolescents with chronic conditions. Soc Indic Res.

[46] Edwards EA, Lumsden J, Rivas C, Steed L, Edwards LA, Thiyagarajan A, Sohanpal R, Caton H, Griffiths CJ, Munafò MR, Taylor S, Walton RT (2016). Gamification for health promotion: Systematic review of behaviour change techniques in smartphone apps. BMJ Open.

[47] Goldsmith RF (2004). Discovering Real Business Requirements for Software Project Success.

[48] Geense WW, van Gaal BG, Knoll JL, Cornelissen EA, Schoonhoven L, Kok G (2016). Online support program for parents of children with a chronic kidney disease using intervention mapping: A development and evaluation protocol. JMIR Res Protoc.

[49] Swallow VM, Knafl K, Santacroce S, Campbell M, Hall AG, Smith T, Carolan I (2014). An interactive health communication application for supporting parents managing childhood long-term conditions: Outcomes of a randomized controlled feasibility trial. JMIR Res Protoc.

[50] Swallow VM, Hall AG, Carolan I, Santacroce S, Webb NJ, Smith T (2014). Designing a Web application to support home-based care of childhood CKD stages 3-5: Qualitative study of family and professional preferences. BMC Nephrol.

[51] Gale NK, Heath G, Cameron E, Rashid S, Redwood S (2013). Using the framework method for the analysis of qualitative data in multi-disciplinary health research. BMC Med Res Methodol.

[52] Swallow V, Carolan I, Smith T, Webb NJ, Knafl K, Santacroce S, Campbell M, Harper-Jones M, Hanif N, Hall A (2016). A novel Interactive Health Communication Application (IHCA) for parents of children with long-term conditions: Development, implementation, and feasibility assessment. Inform Health Soc Care.

[53] Gerson AC, Geary DF, Schaefer F (2016). Psychosocial issues in children with chronic kidney disease. Pediatric Kidney Disease. 2nd edition.

[54] Huby K, Swallow V, Smith T, Carolan I (2017). Children and young people's views on access to a Web-based application to support personal management of long-term conditions: A qualitative study. Child Care Health Dev.

[55] Cantrell KA (2015). Bridging isolation for youth with chronic conditions: Are we thinking virtually?. Pediatr Nurs.

[56] Shah VN, Garg SK (2015). Managing diabetes in the digital age. Clin Diabetes Endocrinol.

[57] Jones R, Cleverly L, Hammersley S, Ashurst E, Pinkney J (2013). Apps and online resources for young people with diabetes: The facts. J Diabetes Nurs.

[58] Whitehead L, Seaton P (2016). The effectiveness of self-management mobile phone and tablet apps in long-term condition management: A systematic review. J Med Internet Res.

[59] Piaget J (1954). The Construction of Reality in the Child.

