# Supplementary material for: Desirable Components for a Customized, Home-Based, Digital Care-Management App for Children and Young People With Long-Term, Chronic Conditions: A Qualitative Exploration
Source: J Med Internet Res. 2017 Jul 4;19(7):e235. doi: 10.2196/jmir.7760 (PMC5516103; doi:10.2196/jmir.7760)
Supplement: Multimedia Appendix 1 [file jmir_v19i7e235_app1.pdf]

Design and usability features suggested by participants
• well designed so users would want to download the app<br>• easy to use (especially for users who have cognitive difficulties)<br>• engaging and useful so they would want to continue using it<br>• exciting and fun<br>• interactive (e.g. swipe/tap screen to get more information)<br>• be very visual<br>• have an audio function for text for users with literacy difficulties<br>• not be too complex<br>• use humour<br>• spell out how to pronounce complex words<br>• avoid acronyms where possible and/or explain commonly used ones<br>• have regular updates/daily ‘bites’ especially in relation to hearing other patient’s stories<br>• for younger children provide opportunities for interaction between the child and their parent/s<br>• be relevant<br>• provide notifications when new content is added to the app<br>• app logo to be distinctive and stand out
